# Symbiotic microbial communities in various locations of the lung cancer respiratory tract along with potential host immunological processes affected

**DOI:** 10.3389/fcimb.2024.1296295

**Published:** 2024-02-02

**Authors:** Jiuling Cheng, Lujia Zhou, Huaqi Wang

**Affiliations:** ^1^ Respiratory Department, The First Affiliated Hospital of Zhengzhou University, Zhengzhou, Henan, China; ^2^ Henan Key Laboratory of Precision Diagnosis of Respiratory Infectious Diseases, The Second Affiliated Hospital of Zhengzhou University, Zhengzhou, Henan, China; ^3^ Zhengzhou Key Laboratory of Precision Diagnosis of Respiratory Infectious Diseases, The Second Affiliated Hospital of Zhengzhou University, Zhengzhou, Henan, China

**Keywords:** lung cancer, respiratory tract, commensal microbiome dysbiosis, immune mechanisms, chromosome aberration

## Abstract

Lung cancer has the highest mortality rate among all cancers worldwide. The 5-year overall survival rate for non-small cell lung cancer (NSCLC) is estimated at around 26%, whereas for small cell lung cancer (SCLC), the survival rate is only approximately 7%. This disease places a significant financial and psychological burden on individuals worldwide. The symbiotic microbiota in the human body has been significantly associated with the occurrence, progression, and prognosis of various diseases, such as asthma, chronic obstructive pulmonary disease (COPD), and cystic fibrosis. Studies have demonstrated that respiratory symbiotic microorganisms and their metabolites play a crucial role in modulating immune function and contributing to the pathophysiology of lung cancer through their interactions with the host. In this review, we provide a comprehensive overview of the microbial characteristics associated with lung cancer, with a focus on the respiratory tract microbiota from different locations, including saliva, sputum, bronchoalveolar lavage fluid (BALF), bronchial brush samples, and tissue. We describe the respiratory tract microbiota’s biodiversity characteristics by anatomical region, elucidating distinct pathological features, staging, metastasis, host chromosomal mutations, immune therapies, and the differentiated symbiotic microbiota under the influence of environmental factors. Our exploration investigates the intrinsic mechanisms linking the microbiota and its host. Furthermore, we have also provided a comprehensive review of the immune mechanisms by which microbiota are implicated in the development of lung cancer. Dysbiosis of the respiratory microbiota can promote or inhibit tumor progression through various mechanisms, including DNA damage and genomic instability, activation and regulation of the innate and adaptive immune systems, and stimulation of epithelial cells leading to the upregulation of carcinogenesis-related pathways.

## 1 Introduction

Globally, lung cancer remains the leading cause of cancer-related deaths, representing a substantial proportion of total cancer fatalities (18.4%) (Siegel et al., 2022). This disease inflicts significant suffering upon patients and imposes a tremendous burden on society. Although the implementation of health education interventions such as smoking cessation programs and early CT screening, along with advancements in immunotherapy, has led to a reduction in mortality rates among lung cancer patients (Howlader et al., 2020; Giaquinto et al., 2022; Xia et al., 2022), the overall five-year survival rate remains low (Society, 2022). The five-year survival rate for NSCLC is 26%, while for SCLC, it is 7%. Furthermore, the specific mechanisms underlying various subtypes of lung cancer remain unclear, and there is still a limited number of therapeutic targets for clinical translation. These factors impose significant burdens on society’s economy and the psychological well-being of patients. Therefore, further exploration of the mechanisms underlying lung cancer and the identification of potential therapeutic targets are of utmost importance.

Traditional beliefs have held that the lower respiratory tract is sterile (Dickson et al., 2016; Huffnagle et al., 2017), as it exhibits lower microbial abundance than other body sites. However, limitations in sampling techniques and the culturing methodology have restricted the identification of bacteria through culture-based methods, resulting in only approximately 1% of bacteria being detectable (Torsvik and Øvreås, 2002; Martiny, 2019). Consequently, many microorganisms remain undetectable (Moffatt and Cookson, 2017). With the continuous development of molecular biology techniques, such as DNA sequencing (Singh et al., 2022), independent of culture-based methods, it has been discovered that the lower respiratory tract of healthy individuals harbors a microbial community. The lung microbiota represents a dynamic assemblage of bacteria, fungi, viruses, and other microorganisms that colonize the lungs through inhalation and different routes (Huxley et al., 1978; Gleeson et al., 1997). Previous research has reported their involvement in the occurrence and progression of various lung diseases, such as asthma, cystic fibrosis, and COPD (Hilty et al., 2010; Silveira et al., 2021; Yagi et al., 2021; Amati et al., 2022; Madapoosi et al., 2022).

The significance of lung microbiota in the occurrence and progression of lung cancer is increasingly recognized by researchers. Previous studies have predominantly focused on investigating the mechanisms underlying gastrointestinal cancers, including gastric cancer and colorectal cancer, concerning the gut microbiota. For instance, Helicobacter pylori (HP) has been identified as a risk factor for gastric cancer (Cheung et al., 2018a; Cheung et al., 2018b; Conteduca et al., 2013), while Fusobacterium nucleatum(Fn) has been implicated in the development of colorectal cancer (Rubinstein et al., 2013; Guo et al., 2020; Liang et al., 2020). Recently, there has been an increasing focus on the lung cancer microbiome. Studies have indicated that the microbial communities in lung cancer patients are dysregulated (Liu et al., 2020; Meng et al., 2023), and specific bacteria or bacterial groups are associated with immune dysregulation in lung cancer (Tsay et al., 2021; Khan et al., 2022). These microorganisms directly or indirectly contribute to the occurrence and development of lung cancer. Extensive research on the lung cancer microbiome highlights its significant role in lung cancer and its potential as a biomarker for early diagnosis and a prognostic indicator for evaluating treatment outcomes.

**Figure 1:**
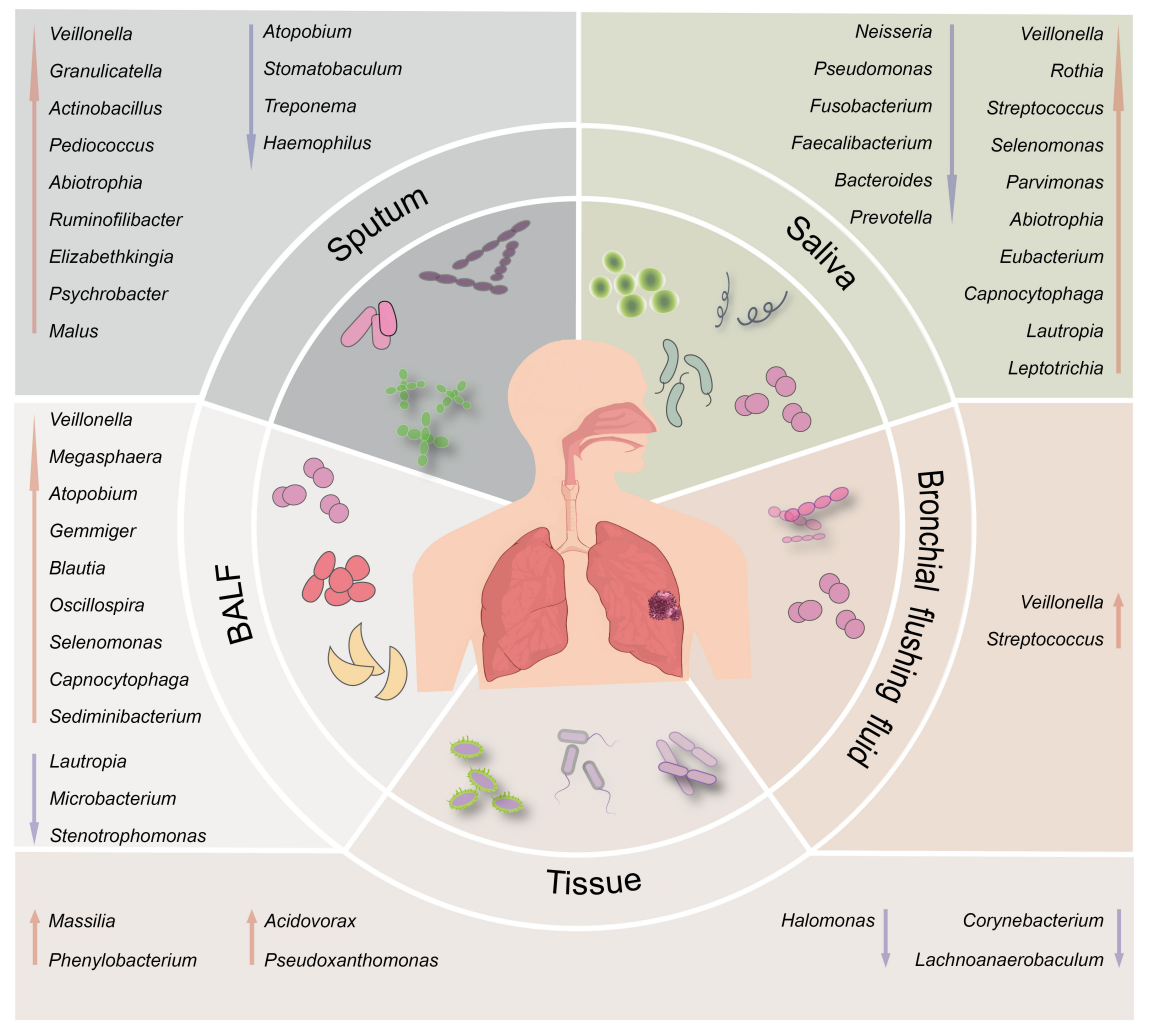
Schematic illustration of the differential respiratory microbiota across different anatomical sites between individuals with lung cancer and healthy subjects. The upward red arrows indicate representative microbiota that exhibit significantly higher abundance in the lung cancer group than the healthy control group. In contrast, the downward blue arrows indicate representative microbiota with decreased abundance.

Currently, comprehensive reviews on the microbial composition in different regions of the lower respiratory tract with lung cancer are lacking. Furthermore, the precise role of specific bacteria in the immune mechanisms of lung cancer remains insufficiently understood. This review provides a comprehensive review of the microbial characteristics in lung cancer patients, focusing on various sample types, including saliva, sputum, BALF, brush samples, and tissue (**Figure 1**). The review further categorizes the microbial features within each sample type based on different factors such as pathological types, metastasis, PD-L1 treatment, smoking, and coal combustion. It explores the potential immune mechanisms by which the microbial community participates in the early diagnosis and treatment of lung cancer, while also revealing the relationship between respiratory microbiota dysbiosis and regulatory factors such as the environment. By offering a more encompassing overview of lung microbiota, this review is a valuable resource for researchers, facilitating further studies and comparisons. Ultimately, it provides a foundation for a better understanding and improvement of the diagnosis and treatment of lung cancer patients.

## 2 The microbiota community in the respiratory tract of lung cancer

### 2.1 Microbiome features of the respiratory tract in lung cancer patients

#### 2.1.1 Differential microbes between lung cancer and healthy controls

##### 2.1.1.1 Upper respiratory tract

Multiple studies on lung cancer currently concentrate on genes with Somatic and Germline variants (Li et al., 2018; Shen et al., 2019). However, genetic factors can only account for 3-15% of hereditary issues (Czene et al., 2002; Dai et al., 2017), while non-genetic environmental factors also play a crucial role, such as respiratory tract symbiotic microbiota (Lee et al., 2016). Although BALF and tissue microbes have considerable research potential, these procedures are invasive. On the other hand, sputum and saliva provide non-invasive means to access respiratory microbiota, making sample collection easier. Therefore, investigating microbiota’s predictive and prognostic value in sputum and saliva is of great significance in understanding the association between microbiota and lung cancer.

Human buccal microorganisms comprise more than 600 bacteria, which play distinct functions under different etiologies (Chen et al., 2010; Dewhirst et al., 2010). In various studies, significant differences have been observed between the bacterial compositions of saliva and sputum samples from individuals with lung cancer compared to healthy controls. Xinmin Yan et al. elucidated the intricate association between the salivary microbial community and lung cancer, presenting a comprehensive account for the first time. They found that the phyla Flavobacteriales, Burkholderiales, Campylobacterales, and Spirochaetales, the family Veillonellaceae, and the genera Capnocytophaga, Selenomonas, and Veillonella are prevalent in lung cancer, although Neisseria is less dominant (Yan et al., 2015).A recent study reported similar findings when comparing saliva samples from lung cancer patients and healthy individuals. The authors observed moderate differences at the phylum level between the two groups. At the genus level, Prevotella_7 (Bacteroidetes), Neisseria (Proteobacteria), Streptococcus (Firmicutes), Veillonella (Firmicutes), and Haemophilus (Proteobacteria) were identified as the most prevalent genera in the oral samples (Sun et al., 2023).

Using recycling free-flow isoelectric focusing (RFFiEF) technology, Xiaoteng Jiang and colleagues are concentrating on analyzing salivary bacteria. Neisseria, Pseudomonas, Serratia, Lactobacillus, and Acinetobacter were more prevalent in healthy subjects, while Rothia, Granulicatella, Parvimonas, Abiotrophia, and Eubacterium significantly increased in the lung cancer group. After the qPCR test, Granulicatella considerably rose, whereas Pseudomonas significantly declined (Jiang et al., 2020).

According to research by Weiquan Zhang et al., the presence of the Firmicutes genera Veillonella, Streptococcus, Lautropia, Leptotrichia, Rothia, and Aggregatibacter was considerably more fabulous in the saliva of NSCLC patients than in the control group. Additionally, the relative abundance of Fusobacterium, Prevotella, Bacteroides, and Faecalibacterium decreased overall in the NSCLC group (Zhang et al., 2019).

Simon J. S. Cameron et al. analyzed sputum samples from ten participants, including four with lung cancer and six without. The lung cancer samples exhibited significantly higher levels of Streptococcus viridans, Granulicatella adiacens, Streptococcus intermedius, and Mycobacterium tuberculosis (Cameron et al., 2017).

Elizaveta Baranova et al. analyzed sputum samples from 80 individuals, consisting of 40 cases with squamous cell carcinoma (LUSC) and 40 healthy controls. Their investigation revealed a significant increase in the phylum Firmicutes, as well as the genera Streptococcus, Bacillus, Gemella, and Haemophilus, including the species Streptococcus agalactiae, among those with squamous cell carcinoma. Intriguingly, no variations in the microbiome were observed to pathological stage or smoking status among lung cancer patients (Baranova et al., 2022).

According to Druzhinin et al., sputum from lung cancer patients exhibited unique taxa, including Actinobacillus, Pediococcus, Abiotrophia, Ruminofilibacter, Elizabethkingia, Psychrobacter, and Malus. Among these, Bergeyella showed a significant increase in lung cancer sputum compared to that of healthy controls. Conversely, the genera Atopobium, Stomatobaculum, and Treponema demonstrated a considerable reduction. The genus Haemophilus was more prevalent in lung cancer, and the species Bergeyella zoohelcum exhibited a considerable increase. Additionally, Atopobium rimae, Treponema amulovorum, and Prevotella (P. histicola and P. sp. oral clone DO014) experienced a considerable decline (Druzhinin et al., 2020).

##### 2.1.1.2 Lower respiratory tract

Lower respiratory tract samples are less susceptible to contamination and are closely associated with lung tissue than saliva and sputum samples. Therefore, studying the correlation between lower respiratory tract bacteria and lung cancer can significantly advance our understanding of this disease’s underlying mechanisms. This section focuses on the microbiological distinctions observed in BALF between lung cancer patients and healthy individuals.

In a study by Wang et al., a comparison was made between BALF and salivary bacteria from lung cancer patients (n = 52) and healthy individuals (n = 15), revealing significant differences. The lung cancer group exhibited considerable variations in the levels of Firmicutes and Fusobacteria in their BALF, as well as significantly different levels of Actinobacteria in their saliva, in comparison to the healthy individuals (Wang et al., 2019).

Identifying potential differences between lung cancer and benign samples using microorganisms can aid in distinguishing between suspicious and confirmed cases. In a study conducted by Sang Hoon Lee and colleagues, they examined 28 BALF samples, including 20 from lung cancer patients and eight from individuals with benign illnesses. The researchers found a significant elevation of Veillonella, Megasphaera, Atopobium, and Selenomonas in the lung cancer group (Lee et al., 2016).

The sample size was expanded to include 54 individuals, comprising 32 lung cancer patients and 22 individuals with benign lung disease. The results of this study revealed significant increases in six genera (c: TM7-3, Capnocytophaga, Sediminibacterium, Gemmiger, Blautia, and Oscillospira), while four genera (Microbacterium, Stenotrophomonas, Lautropia, and f: Pseudonadaceae) exhibited significant decreases. These ten genera and three tumor markers (CEA, NSE, and CYFRA21-1) were used to construct a random forest model. The model demonstrated promising predictive capabilities, with an area under the curve (AUC) value of 84.52% for lung cancer, indicating its potential for clinical application (Cheng et al., 2020).

Wen Zeng et al. conducted a study involving BALF samples from 46 NSCLC patients and 29 individuals with benign illnesses. The researchers found that Prevotella, Alloprevotella, and Veillonella were significantly elevated in the lung cancer group. Additionally, positive associations were observed between Prevotella and Megasphaera, Alloprevotella, and Actinomyces. Subcutaneous transplantation and endotracheal instillation experiments were conducted to further investigate the impact of Veillonella parvula. The results showed that subcutaneous transplantation significantly promoted lung cancer, while endotracheal instillation did not lead to tumor formation. These findings suggest that the influence of microorganisms on lung cancer may vary in terms of chronicity, duration, or adequacy. These numerous genera could serve as diagnostic indicators and therapeutic targets (Zeng et al., 2022). For brushing samples, lung cancer patients have a higher abundance of Veillonella and Streptococcus compared to healthy individuals (Tsay et al., 2018). However, it is essential to note that different investigations have reported varying bacterial compositions, which could be attributed to the limited sample sizes. Hence, these findings should be validated through large-scale studies.

##### 2.1.1.3 The biodiversity

The diversity of sputum microorganisms was found to be lower in lung cancer patients compared to healthy controls (Hosgood et al., 2014; Hosgood et al., 2019; Lu et al., 2021). However, V.G. Druzhinin et al. reported no significant change in the diversity of sputum microorganisms between lung cancer patients and healthy individuals (Druzhinin et al., 2020; Druzhinin et al., 2021). These studies highlight notable disparities in β diversity. Furthermore, Salvador Bello et al. found no difference in the α diversity of salivary microbes between lung cancer patients and healthy individuals. Still they observed a substantial difference in the β diversity of salivary bacteria (Bello et al., 2021). However, Weiquan Zhang et al. have presented arguments highlighting significant variations in both α diversity and β diversity between the two groups when considering saliva (Zhang et al., 2019).

There are conflicting findings regarding the microbial diversity in BALF observed in different studies. Sílvia Gomes, Wen Zeng et al. found that the BALF microbial α-diversity in lung cancer patients was higher than in non-lung cancer patients (Gomes et al., 2019; Zeng et al., 2022). However, Sang Hoon Lee et al. reached the opposite conclusion (Lee et al., 2016). Several other studies have also reported no significant differences in microbial α- and β-diversity between lung cancer and benign disease groups in BALF (Tsay et al., 2018; Wang et al., 2019; Cheng et al., 2020; Zhuo et al., 2020). However, Sang Hoon Lee et al. suggested a significant difference in β-diversity between cancer and benign control groups (Cheng et al., 2020; Zeng et al., 2022).

#### 2.1.2 Microbiome under different pathological types

##### 2.1.2.1 Upper respiratory tract

It is essential to distinguish different pathologic types after comparing overall lung cancer with healthy controls. A comparative analysis of salivary microbes in LUAD and LUSC revealed distinct patterns. Specifically, in the saliva of squamous cell carcinoma, Streptococcus and Porphyromonas were significantly lower, while Prevotella showed no significant changes between the two groups (Yan et al., 2015). On the other hand, Streptococcus, Capnocytophaga, and Actinomyces were enriched in the saliva of lung cancer patients. The LSCC group demonstrated a higher enrichment of Capnocytophaga and Actinomyces than the healthy group. Additionally, the SCLC group exhibited a higher concentration of Streptococcus, while Rothia was significantly higher in the adenocarcinoma group (Wang et al., 2019).

In a study conducted by Qixin Leng et al., 25 bacteria in adenocarcinoma and squamous cell carcinoma sputum and tissues were compared using ddPCR. The results revealed significant increases in Capnocytophaga in adenocarcinoma samples and Acidovorax in squamous cell carcinoma samples, with both sputum and tissue demonstrating similar trends. However, adenocarcinoma tissues exhibited exclusive elevations of Haemophilus and Fusobacterium, contradicting previous research indicating Haemophilus accumulation in lung cancer sputum (Lu et al., 2021). Squamous cell carcinoma sputum, on the other hand, contained only Streptococcus and Veillonella. Also, Helicobacter levels were reduced solely in adenocarcinoma sputum (Leng et al., 2021).

Danhui Huang et al. investigated the microbiological variations between sputum and bronchial flushing fluid (BWF) samples and reported the microorganisms of squamous cell cancer and adenocarcinoma, respectively. The levels of Streptococcus (Firmicutes) in sputum were higher in lung cancer patients than in BWF (benign lung disease with fibrosis). Moreover, peripheral lung cancer exhibited higher levels of Streptococcus (Firmicutes) in sputum than central lung adenocarcinoma (Huang et al., 2019), which contradicts earlier research (Yan et al., 2015). Additionally, large-cell carcinoma demonstrated higher levels of Veillonella and Leptotrichia than adenocarcinoma (Druzhinin et al., 2020). Regarding sputum α microbial diversity, no significant differences were found between lung adenocarcinoma and lung squamous cell carcinoma (Huang et al., 2019).

##### 2.1.2.2 Lower respiratory tract

A recent study showed that the populations of Haemophilus parainfluenza, Neisseria subflava, Porphyromonas endodontalis, and Fusobacterium nucleatum were significantly higher in the adenocarcinoma group when compared to squamous cell carcinoma (Jang et al., 2023). In the study conducted by Ke Wang et al., it was found that Veillonella and Capnocellhaga exhibited significantly higher levels in the BALF of patients with lung squamous cell carcinoma (LUSC). Conversely, Lactobacillus showed a marked increase in the BALF of individuals with small-cell lung carcinoma (SCLC) (Wang et al., 2019).

In the study conducted by Minglei Zhuo et al., significant increases in the genera Spiroplasma and Weissella were observed in the BALF of the lung cancer group when compared to the healthy side of the lung affected by cancer (Zhuo et al., 2020). Furthermore, Slvia Gomes et al. examined the BALF samples from individuals with adenocarcinoma, squamous cell carcinoma, and healthy controls. They also used RNA sequencing data from the TCGA database to validate their findings. Sphingomonas, Brevundimonas, Acinetobacter, and Methylobacterium were exclusively found in lung adenocarcinoma (LUAD), while Enterobacter, Morganella, Kluyvera, and Capnocytophaga were predominantly present in lung squamous cell carcinoma (LUSC). The prevalence of a proteobacteria-dominated microbiome in the BALF of squamous cell carcinoma is associated with a progressive decline in survival rates (Gomes et al., 2019).

Danhui Huang et al. conducted a study to identify the microbiological colonies in squamous cell carcinoma and adenocarcinoma and the microbial differences between sputum and bronchial washing fluid (BWF) samples in lung cancer patients. The results showed that BWF samples exhibited a significantly higher abundance of Proteobacteria and a lower abundance of Firmicutes than sputum. Comparing the bronchial washing fluids (BWFs) across various pathological types, Firmicutes and the genera Veillonella, Megasphaera, Actinomyces, and Arthrobacter were found to be more prevalent in NSCLC with no distant metastasis (AD-M0) than in squamous cell carcinoma with no distant metastasis (SC-M0). Conversely, Capnocytophaga and Rothia showed a significant decrease in AD-M1 compared to SC-M1, and no association was observed between these distinct bacteria and smoking status. AD-M1 demonstrated a lower abundance of Firmicutes and Streptococcus in BWF than SCC-M1. Furthermore, SCC-M1 exhibited a higher presence of Veillonella and Rothia than SCC-M0 (Huang et al., 2019).

There is also controversy regarding the diversity comparison among different pathological types. Danhui Huang et al. suggested that microbial diversity is not associated with pathological types (Huang et al., 2019), while Sílvia Gomes et al. found that the α-diversity of microbiota was higher in squamous cell carcinoma compared to adenocarcinoma (Gomes et al., 2019). Interestingly, Erin A Marshall et al. found, through a 10-year follow-up, that there was no significant difference in α-diversity between individuals with a previous history of lung cancer, those who would develop lung cancer in the future, and non-lung cancer patients (Marshall et al., 2022).

#### 2.1.3 The microbiota community associated with lung cancer metastasis

Metastasis of lung cancer is a substantial contributor to death and recurrence in NSCLC (Siegel et al., 2022). Several studies have revealed that microbes play a role in tumor metastasis. In a survey conducted by Hui Lu et al., a comparison of microbes in feces and sputum was performed among 121 participants, including 87 with NSCLC and 34 in good health. The findings revealed that gut microbes were exclusively associated with lung cancer that did not metastasize, whereas sputum microbes showed a connection to lung cancer regardless of metastasis. Higher concentrations of Haemophilus (specifically, Haemophilus parainfluenzae and Haemophilus influenzae) were observed in lung cancer samples compared to healthy controls. Additionally, sputum and feces from individuals with metastatic lung cancer exhibited enrichment of Coriobacteriaceae and Actinomyces. The sputum of advanced metastatic lung cancer showed a higher abundance of Pseudomonas, while Campylobacter was more abundant in both the regular group and advanced metastatic lung cancer. An analysis of a random forest model determined that the sputum model provided better predictive accuracy than intestinal microbes. Hence, sputum microorganisms were more closely associated with the development of lung cancer than intestinal microbes (Lu et al., 2021).

Dan Hui Huang et al. examined 85 sputum samples obtained from newly diagnosed patients with NSCLC. Their analysis revealed that in early-stage lung cancer (stage I and stage II), the phylum Firmicutes and the genera Peptoniphilus, Granulicatella, Hylemonella, Actinobacillus, SMB53, and Gemella were more abundant. On the other hand, in advanced-stage lung cancer (stage III and stage IV), the phyla Actinobacteria and the genus Actinomyces exhibited more significant enrichment. Compared to the non-metastatic group, the metastatic group revealed significant enrichment of the genera Peptostreptococcus, Peptococcus, Parabacteroides, and Escherichia. Moreover, in the lymph node metastasis group, there was a considerable increase in the genera Parvimonas and Pseudomonas. The EGFR mutant group exhibited significant enrichment of Bacteroidetes and Tenericutes, along with the genera Sharpea, Prevotella, Porphyromonas, Parvimonas, Desulfovibrio, Mycoplasma, Actinobacillus, Dialister, and Eikenella. Compared to stage III lung cancer, only Paludibacter showed a significant increase. The specific airway genus and metabolic function of sputum microbiota were found to undergo changes associated with tumor stage, intrathoracic metastasis, lymph node metastasis, and EGFR mutation (Huang et al., 2022). Porphyromonas endodontalis was significantly more abundant in the sputum of patients with stages I-II than those with stages III-IV. Additionally, patients with metastases exhibited higher levels of Capnocytophaga and lower levels of Atopobium rimae than those without metastasis (Druzhinin et al., 2020).

Significant differences in microbial diversity were observed in sputum samples when comparing lung cancer’s early and advanced stages.However, microbial diversity was found to be unrelated to stages III and IV, intrathoracic and lymph node metastases, and EGFR mutation (Huang et al., 2022).

### 2.2 The relationship between microbiota and the host genome

Lung microbes and genes collaborate to facilitate the onset and progression of lung cancer. In their study, V. G. Druzhinin et al. investigated sputum samples obtained from lung cancer patients and controls. They focused on exploring the connection between microbes and the occurrence of chromosomal mutations. Interestingly, their findings revealed a significant decrease in the genus Atopobium, while Alloprevotella exhibited a significant increase among patients with a high frequency of chromosomal aberrations (CA) (Druzhinin et al., 2020). Moreover, V. G. Druzhinin et al. expanded the sample size to include 66 lung cancer patients and 62 healthy subjects. Their investigation revealed significant increases in Streptococcus, Bacillus, Gemella, and Haemophilus among lung cancer patients. Additionally, they found that Bacteroides, Lachnoanaerobaculum, Porphyromonas, Mycoplasma, and Fusobacterium were associated with the frequency of chromosomal aberrations. Interestingly, the genera Megasphaera and Selenomonas bovis negatively correlated with micronuclei (MN) (Druzhinin et al., 2021).

### 2.3 The relationship between microbiota and environmental factors

#### 2.3.1 Upper respiratory tract

Lung microorganisms can be influenced by environmental factors such as coal combustion and smoking, which have implications for the development and progression of lung cancer. In his study, H. Dean Hosgood, III examined the impact of microbiota in sputum and buccal samples on lung cancer among never-smoking women. Additionally, the study explored the effect of coal on these associations. The findings revealed that the diversity of bacterial communities in buccal samples was comparable between the case and control groups. However, significant differences were observed in sputum samples. Specifically, Granulicatella, Abiotrophia, and Streptococcus were abundant in lung cancer patients’ sputum. Lung microorganisms can be influenced by environmental factors such as coal combustion and smoking, which have implications for the development and progression of lung cancer. In his study, H. Dean Hosgood, III examined the impact of microbiota in sputum and buccal samples on lung cancer among never-smoking women.

Additionally, the study explored the effect of coal on these associations. The findings revealed that the diversity of bacterial communities in buccal samples was comparable between the case and control groups. However, significant differences were observed in sputum samples. Specifically, Granulicatella, Abiotrophia, and Streptococcus were abundant in lung cancer patients’ sputum. In smoking-related lung cancer patients, the species Selenomonas bovis, genus Bacteroides, and genus Selenomonas were found to be more abundant compared to never-smoking lung cancer patients.

Conversely, the genus Peptostreptococcus exhibited higher abundance in never-smoking lung cancer patients. Among healthy smokers, the species Bulleidia moorei, genus Granulicatella, and genus Bulleidea were prevalent, whereas the genus Neisseria was abundant in healthy nonsmokers (Druzhinin et al., 2020). For more detailed information, please refer to **Table 1**.

**Table 1:** Summary of studies of lung cancer-associated microbiome in saliva and sputum.

Year	Reference PMID	Sample Type	Sample Size	Subgroup	Analytical Method (hypervariable regions)	Main Findings
2019	31598405	BWF and sputum	92	BWF: LUAD(n=21), LUSC(n=19) sputum: LUAD(n=37), LUSC(n=15)	16S rRNA sequencing (V3-V4)	1. compared BWF, Streptococcus (Firmicutes) in sputum is higher. 2. Streptococcus in peripheral LUAD was significantly higher than that of central lung adenocarcinoma in the sputum group
2015	26693063	saliva	86	discovery cohort: SCC(n=10), AD(n=10), control(n=10) validation cohort: SCC(n=13), AD(n=28), control(n=15)	16S rDNA sequencing (V3-V6)	1. Phyla Flavobacteriales, Burkholderiales, Campylobacterales, Spirochaetales, and genus Capnocytophaga, Selenomonas, and Veillonella in lung cancer were higher, while phylum Bacteroidales and Neisseria were lower. 2. Streptococcus and Porphyromonas were significantly decreased in the saliva of squamous cell carcinoma
2021	34787462	sputum and stool	121	NSCLC(n=87), health(n=34)	16S rRNA sequencing	1. Compared with the healthy group, Haemophilus was enriched in the lung cancer group. 2. the sputum and feces of the family Coriobacteriaceae and genus Actinomyces were enriched in the metastatic lung cancer group; 3. Pseudomonas had a high abundance in advanced metastatic lung cancer sputum.
2021	33673596	tissue and sputum	237	cohort 1: tissue: tumor (n=31) and paired tissue(n=31); sputum: cancer(n=17), healthy smokers(n=10) validation cohort: sputum:cancer(n=69), healthy smokers(n=79)	ddPCR	1. Acidovorax is overexpressed in squamous cell carcinoma tissues compared to non-squamous and adenocarcinoma tissues. 2. Capnocytophaga was overexpressed in adenocarcinoma tissues, and Haemophilus and Fusobacterium were lower than those in squamous cell carcinoma or paracancerous tissue. 3. Acidovorax, Streptococcus, and Veillonella were overexpressed in the sputum of squamous cell carcinoma, while Helicobacter was underexpressed.
2019	30942501	sputum	90	never smoking smoking: cancer(n=45), control(n=45)	16S rRNA sequencing (V1-V2)	The higher risk of lung cancer is associated with reduced levels of phylum Fusobacteria
2014	24895247	sputum and buccal samples	16	never smoking female: cancer(n=8), control(n=8)	16S rRNA sequencing (V1-V2)	1. Granulicatella, Abiotrophia and Streptococcus in sputum were enriched in lung cancer. 2. Proteobacteria (Neisseria) were enriched in Reshui Village (smoky coal). 3. Bacilli and Streptococcus (tentatively assigned to S. infantis and S. anginosus) were enriched in Laibin Town (smokeless coal). 4. Bacilli species (Streptococcus infantis and Streptococcus anginosus) were enriched in PAH-rich lung cancer samples
2022	35142041	sputum	85	NSCLC:early stage(n=22), advanced stage(n=51), unidentified(n=12)	16S rRNA sequencing (V3-V4)	1. IPaludibacter in stage IV was significantly higher than in stage III. 2. The early stage significantly enriched Firmicutes, genera Peptoniphilus, Granulicatella, Hylemonella, Actinobacillus, SMB53, and Gemella. 3. The AS group significantly enriched the phylum Actinobacteria and genus Actinomyces.
2020	32541778	sputum	34	lung cancer(n=17), control(n=17)	16S rRNA sequencing (V3-V4)	1. The unique genera of lung cancer are Actinobacillus, Pediococcus, Abiotrophia, Ruminofilibacter, Elizabethkingia, Psychrobacter, and Malus. 2. Compared with healthy controls, Bergeyella was significantly increased in lung cancer patients’ sputum. Genus Haemophilus was more likely to appear in patients with lung cancer, and Bergeyella zoohelcum was significantly increased. 3. Compared with non-smoking lung cancer, Selenomonas bovis, genera Bacteroides, and Selenomonas are more prevalent in smoking patients, while Peptostreptococcus Zhouea are more prevalent in non-smoking patients. 4. Veillonella and Leptotrichia are higher in large cell carcinoma than LUAD
2017	28542458	sputum	10	lung cancer(n=4), control(n=6)	16S rRNA sequencing	Streptococcus viridans, Granulicatella adiacens, Streptococcus intermedius, and Mycobacterium tuberculosis were significantly higher in lung cancer samples.
2022	36143401	sputum	80	LUSC(n=40), control(n=40)	metagenomic sequencing	1. Firmicutes, Streptococcus, Bacillus, Gemella, and Haemophilus, species Streptococcus agalactiae, were significantly increased in squamous cell carcinoma. 2. There is no difference between stage and smoking for lung cancer patients
2021	33454779	sputum	128	lung cancer(n=66), control(n=62)	16S rRNA sequencing (V3-V4)	1. There were significant increases in Streptococcus, Bacillus, Gemella, and Haemophilus in lung cancer 2. Bacteroides, Lachnoanaerobaculum, Porphyromonas, Mycoplasma, and Fusobacterium are related to chromosome mutations in patients with lung cancer. 3. Megasphaera genera and Selenomonas bovis increase
2020	32786473	saliva	43	lung cancer(n=22), control(n=21)	rFFiEF	1. six bacterial genera were significantly up-regulated in the lung cancer group, while two were down-regulated. 2. qPCR confirmed that Granulicatella increased significantly in the lung cancer group, while Pseudomonas decreased significantly
2021	35699005	bronchial biopsies, saliva, and faecal samples	41	central lung cancer(n=25), control(n=16)	16S rDNA sequencing (V3-V4)	Streptococcus, Rothia, Gemella, and Lactobacillus abundances can distinguish patients’ saliva from controls.
2019	31205521	saliva	59	NSCLC(n=39), control(n=20)	16S rRNA sequencing (V1-V2)	1. Firmicutes and Veillonella, Streptococcus, Lautropia, Leptotrichia, Rothia, and Aggregatibacter in saliva of the NSCLC group was significantly higher than that of the control group. 2. The relative abundance of Fusobacterium, Prevotella, Bacteroides, and Faecalibacterium decreased in the NSCLC group

#### 2.3.2 Lower respiratory tract

Lung cancer and smoking are widely acknowledged to be closely related. However, only a small percentage of smokers, approximately 10-15%, will develop lung cancer. This presents a clinical challenge in predicting which smokers are at risk (Bruder et al., 2018; Herbst et al., 2018). Moreover, smoking has been shown to impair the function of the epithelial barrier, increasing the likelihood of lung resident bacteria infiltrating the lungs and contributing to the onset and progression of disease (Hou et al., 2019; Wong et al., 2020). Therefore, a comprehensive exploration of the interplay between smoking and lung bacteria has the potential to enhance the early detection of lung cancer.

Erin A Marshall et al. conducted a study where 400 bronchial brush samples were subjected to 16S microbiota sequencing. The samples were divided into an exploration group and a validation group. Over 10 years, the participants were closely monitored, and differentiated microbial communities were analyzed using linear differentiation analysis and the establishment of a linear model. The results revealed that patients with higher scores had a greater risk of developing lung cancer, and the onset occurred earlier, indicating the potential of microbiota in predicting the occurrence of lung cancer (Marshall et al., 2022).

Jun-Chieh J Tsay et al. compared oral and bronchial brush samples from individuals with lung cancer (n=39), benign lung nodules (n=36), and healthy individuals (n=10). They found that the lung cancer group exhibited an abundance of Streptococcus and Veillonella, the benign lung nodule group showed enrichment of Streptophyta, Moraxellaceae, and Stenotrophomonas. At the same time, the samples from healthy individuals were enriched with Acholeplasma and Acidocella (Tsay et al., 2018). Please refer to **Table 2** for more details.

**Table 2:** Summary of studies of lung cancer-associated microbiome in lower respiratory tract.

Year	Reference PMID	Sample Type	Sample size	Subgroup	Analytical Method (hypervariable regions)	Main Findings
2019	31598405	Bronchial washing fluid and sputum	92	1. BWF(n=40) and sputum(n=52) 2. SCC-M0(n=7) vs AD-M0(n=7), SCC-M1(n=7) vs AD-M1(n=12)	16S rRNA sequencing (V3-V4)	1. Phylum Firmicutes and genera Veillonella, Megasphaera, Actinomyces, and Arthrobacter in AD-M0 were significantly higher than SCC-M0.2. Capnocytophaga and Rothia in AD-M1 are substantially lower than SC-M1.3. In BWF, Phylum Firmicutes and genera Streptococcus AD -M1 are markedly lower than AD-M0 4. Veillonella and Rothia in SCC -M1 were considerably higher than SCC-M0。
2022	35255902	Bronchial washing fluid	400	1. Cohort 1 (n=230): incident-(n=36), prevalent- (n=12), and no-cancer (n=182) 2. Cohort 2 (n=115): incident-(n=18), prevalent- (n=6), and no-cancer (n=91) 3. Cohort 3 (n=48): incident- (n=5), prevalent- (n=3), no-cancer (n=40)	16S rDNA sequencing, V4	the lung cancer group was strongly correlated with the Bacilli class, Lactobacillales, the Streptococcus genus and its family, and the Paenibacillus genus and its family
2016	27987594	BALF	28	lung cancer(n=20), benign diseases(n=8)	16S rRNA sequencing (V1-V3)	The phylum Firmicutes and TM7, genera (Veillonella, Megasphaera, Atopobium, and Selenomonas) are significantly increased in lung cancer; the abundance of Streptococcus, Alloprevotella, and Porphyromonas was higher in lung cancer patients with a history of smoking
2019	30994108	BALF and saliva	66	lung cancer(n=51), health control(n=15)	16S rDNA sequencing, V4	1. The BALF of LUAD and SCLC microbes at the phylum level are mainly Firmicutes, and at the genus level, they are mainly Pseudomonas. 2. LUSC: mainly Veillonella and Corynebacterium,
2020	32676331	BALF	54	lung cancer(n=32), benign diseases(n=22)	16S rRNA sequencing (V3-V4)	1. lung cancer group vs. benign disease, phylum TM7, six genera (c: TM7-3, Capnocytophaga, Sediminibacterium, Gemmiger, Blautia, and Oscillospira) increased significantly. Phylum Proteobacteria, four genera Microbacterium, Stenotrophomonas, Lautropia, and f: Pseudomonadaceae decreased significantly. 2. Capnocytophaga, Sediminibacterium and c:TM7-3 was significantly correlated with CEA and CYFRA21-1.
2021	34963470	BALF	84	low expression of PD-L1(n=59), high expression of PD-L1(n=24)	16S rRNA sequencing (V3-V4)	1. The phylum Firmicutes and the genus Veillonella dispar (Firmicutes) were significantly higher in the PD-L1 high group, and the phylum Proteobacteria and the genus Neisseria (Proteobacteria) were significantly higher in the PD-L1 low group. 2. phylum Proteobacteria and Bacteroidetes, genus Haemophilus, species Haemophilus influenzae and Neisseria perflava were higher in responders than nonresponders, while genus Veillonella and V. dispar were higher in the responder group
2022	35389889	BALF and PBMC	12	responders for ICIs(n=6), nonresponders(n=6)	16S rRNA sequencing (V3-V4)	Proteobacteria in responders is lower, while Bacteroidetesis is higher than in nonresponders.
2020	32984019	BALF	100	paired samples from cancerous lung(n=50) and the contralateral non-cancerous lung(n=50)	16S rRNA sequencing	phylum Tenericutes, genus Spiroplasma, and genus Weissella increased significantly in the lung cancer group, but phylum Bacteroidetes decreased significantly
2022	35254206	BALF	75	NSCLC(n=46), benign disease(n=29)	16S rRNA sequencing (V3-V4)	1. Prevotella, Alloprevotella, and Veillonella in lung cancer were significantly increased 2. Prevotella and Megasphaera, Alloprevotella and Actinomyces are positively correlated.
2018	29864375	Airway brushings	85	lung cancer(n=39), benign pulmonary nodule(n=36), and health(n=10)	16S rRNA sequencing, V4	1. Streptococcus and Veillonella are enriched in lung cancer.2. Streptophyta, Moraxellaceae, and Stenotrophomonas are enriched in benign pulmonary nodule.3. Acholeplasma and Acidocella are enriched in health
2019	31492894	BALF	103	1. lung cancer(n=49), control(n=54) 2. TCGA: ADC(n=515) and SCC(n=501)	16S rRNA sequencing (V3-V6)	1. Sphingomonas, Brevundimonas, Acinetobacter, and Methylobacterium are only in LUAD. 2. Enterobacter, Morganella, Kluyvera, and Capnocytophaga are mainly in LUSC. 3. Clusters of p _ C1 (Proteobacteria dominate) in SCC cases appear to be associated with a slow decline in survival rate

## 3 The association between intratumoral microbiota and lung cancer

### 3.1 The intratumoral microbiota characteristics in lung cancer

Studies have described on the association between bacteria in alveolar lavage fluid, lung brush samples, sputum, and saliva and lung cancer (Hilty et al., 2010; Charlson et al., 2011; Erb-Downward et al., 2011; Pragman et al., 2012; Borewicz et al., 2013; Segal et al., 2013; Willner et al., 2013; Dickson et al., 2014; Bassis et al., 2015; Dickson et al., 2015), but due to the invasiveness of lung tissue, difficult to get samples. The low microbial abundance, there are few microorganism studies in lung cancer tissues.

The location of the tumor correlates with its aggressiveness, and the 5-year survival rate after pulmonary lobectomy in the lower lobe is poorer than that of the tumor in the upper lobe (Hayakawa et al., 1996; Inoue et al., 2004; Ou et al., 2007). Rea Bingula et al. analyzed 18 cases of NSCLC saliva, BALF (directly obtained from the resected lung lobe), cancerous tissue, paracancerous tissue, and distant cancer tissue and discovered that the microbiomes in saliva, BALF, and tissue were distinct. The tissues were dominated by Proteobacteria, while BALF and saliva were dominated by Firmicutes. All samples showed an increase in the abundance of Firmicutes in lower lobe tumors and a decrease in Proteobacteria. In addition, depending on the location of the tumor, Actinobacteria and Flavobacteriia have opposing abundances in BAL and extratumor tissues. Although tumor microbiota appears to be least impacted by location, paracancerous tissues exhibit the most susceptibility, with a considerable increase in resemblance to BAL microbiota in the upper lobe (Bingula et al., 2020). See **Table 3** for details.

**Table 3:** Summary of studies of lung cancer-associated microbiome in Lung tissues.

Year	Reference PMID	Sample Type	Sample size	Subgroup	Analytical Method (hypervariable regions)	Main Findings
2016	27468850	lung tissues	196	tumor(n=31), the area distant from the tumor(n=165)	16S rRNA sequencing, (V3–V5)	1. More proteobacteria, Thermi, and Cyanobacteria exist in the lung tissues than in other body parts. 2. Compared with patients without metastasis, the relative abundance of Proteobacteria in remote cancer tissues of patients with metastasis has significantly increased
2018	30323970	lung tissues	40	emphysema(n=10),tumor(n=11) and both(n=19)	16S rRNA sequencing (V4)	1. Firmicutes and Bacteroidetes increased significantly in the lung cancer group. At the genus level, Prevotella (Bacteroidetes), Bifidobacterium (Actinobacteria), Proteobacteria (primary the genera) of Streptococcus (Firmicutes) and Escherichia/Shigella and Haemophilus (Proteobacteria). 2. Acinetobacter and Acidovorax increased significantly in the emphysema group
2021	32340803	lung tissues, BALF, and saliva	48	recurrence(n=18), no recurrence(n=18)	16S rRNA sequencing, (V3-V4) and RNA Seq	1. Among saliva, Delftia and Bifidobacterium genera are twice as high in patients with recurrence as in non-recurrence patients. 2. The abundance of Staphylococcus was higher in patients with recurrence of lung cancer tissues, and the abundance of Bacillus and Anaerobacillus was lower. There was no difference in non-lung cancer tissue between the two groups. 3. There were differences in the 19 genera between the two groups in BALF.
2022	35479075	lung tissues	53	high-IAP(n=17), low IAP(N=17) and FLC(n=19)	16S rRNA sequencing and RNA-seq	The enrichment microorganisms are divided into opportunistic pathogens, probiotics, and microorganisms that degrade pollutants. The third category involves Sphingomonas, Sphingopyxis, etc., which are helpful for degrading pollutants but may also lead to epithelial damage and chronic inflammation.
2022	35693079	lung tissues	216	AC EGFR+(n=54), AC EGFR-(n=54), SCC (n=54) and adjacent normal tissues(n=54)	16S rRNA sequencing	Stenotrophomonas accounted for most of the NSCLC tissues with recurrence, and Haemophilus influenzae (H. influenzae) was enriched in patients with squamous cell carcinoma.
2022	36091439	lung tissues, BALF	74	diseased BALF(n=11), paired controlvBALF (n=11), GGO(n=26) and distant control (n=26)	16S rRNA sequencing, (V4/V3/V3-V4/V4-V5)	1. In BALF, Rothia were higher on the healthy side, Faecalibacterium prausnitzii and Bacteroides were higher on the GGO side. 2. At the phylum level, Proteobacteria have a significant difference in tumor tissue and adjacent tumor tissue.
2022	36303210	lung tissues	80	tumor(n=39), the area distant from the tumor(n=41)	16S rRNA sequencing (V4)	1. In tumor tissue, higher abundance of Pseudomonadales, Actinomycetales, and Marmoricola aurantiacus species was associated with poorer survival, especially DFS. 2. the higher abundance of Bacteroidia and Clostridia and Bacteroidales and Clostridiales is correlated with the lower survival rate in normal lung tissues.
2020	32450847	lung tissues, BALF, and saliva	79	Upper lobe T(n=10), Lower lobe T(n=8)/saliva(n=17), BAL(n=15), T(n=16), PT(n=14), DT(n=17)	16S rRNA sequencing (V3-V4)	1. Proteobacteria were dominant in tissue samples, while firmicutes increased in the BAL and saliva, class Clostridia and Bacilli, respectively. 2. All samples showed an increased abundance of Firmicutes in the upper lobe of the tumor. However, Proteobacteria decreased. 3. clades Actinobacteria and Flavobacteriia showed opposite abundance between the BAL and peritumoral tissues, depending on the location of the lung lobes.
2020	30733306	lung tissues	38	tumor(n=19), the area distant from the tumor(n=19)	16S rRNA sequencing (V4)	1. In tumor tissues, families Koribacteraceae and Lachnospiraceae were associated with reduced recurrence and disease-free survival. 2. lung tumor tissues had a higher Veillonellaceae family abundance. There is a lower abundance of the Cloaciobacter genus and a lower abundance of the Erysipelotrichaceae family.
2021	33891617	lung tissues	58	tumor(n=29), the area distant from the tumor(n=29)	16S rRNA sequencing (V3-V4)	There are no significant differences in bacteria among groups.
2020	32933105	lung tissues	178	tumor(n=89), the area distant from the tumor(n=89)	16S rRNA sequencing (V3-V4)	1. The content of gram-positive bacteria was significantly increased in LUAD. 2. The combination of high bacterial load and increased iNOS expression in the tumor was a favorable prognostic symptom factor, while the combination of high bacterial load and increased FOXP3^+^ cell number was a marker of poor prognosis.
2018	30127774	FFPE	29	LUAD(n=11), LUSC(n=10), adjacent normal FFPE samples(n=8)	16S rRNA sequencing	1. The comparison of Actinutes and Bacteriodetes in NSCLC tissues significantly differs from that in normal tissues. 2. Firmicutes and Proteobacteria are significantly lower in adenocarcinoma than in squamous cell carcinoma, and Cyanobacteria are significantly higher in adenocarcinoma. Melainabacteria is only found in squamous cell carcinoma
2022	35005565	lung tissues	286	tumor(n=143), the area distant from the tumor(n=143)	16S rRNA sequencing (V3-V4)	1. Massilia, Phenylobacterium, and Pseudoxanthomonas are mainly distributed in tumor tissues, while Brevibacillus, Cupriavidus, and Anaerococcus are more abundant in non-tumor tissues. 2. Brevundimonas, Ruminococcus, and Polaromonas differed between squamous cell carcinoma and adenocarcinoma. 3. More Massilia and Sphingobacterium and less Acidovorax in the tumor tissues of smokers 4. Acidovorax and Massilia were rich in TP53 mutation-positive tumors.
2018	30143034	lung tissues	336	tumor(n=143), the area distant from the tumor(n=144), control(n=49) TCGA: tumor(n=974), normal adjacent(n=108)	16S rDNA sequencing (V3)	Acidovorax showed a higher abundance in squamous cell carcinoma tissues with TP53 mutations

The diversity of microorganisms reflects the complexity of the entire microbiome, and existing research has studied the diversity of microorganisms in lung tissue from various subgroups. The diversity of tumor tissue is less than that of paired distant cancer tissues (Yu et al., 2016; Peters et al., 2019; Chen et al., 2022; Kim et al., 2022), Advanced lung cancer has less diversity than early lung cancer, which has less diversity than normal tissue (Kim et al., 2022), benign lung tissue is less diverse than cancer tissue (Chen et al., 2022), and the diversity of lung tissue in healthy individuals is greater than that in cancer patients, regardless of tumor tissue or tumor-adjacent tissue (Greathouse et al., 2018). The diversity of lung tissue in healthy individuals is greater than that in cancer patients (Bingula et al., 2020; Kovaleva et al., 2020; Dong et al., 2022; Peters et al., 2022), and there is no significant difference between adenocarcinoma and squamous cell carcinoma tissues (Apopa et al., 2018; Kovaleva et al., 2020). Nathan Dumont-Leblond and colleagues hypothesized that the α diversity of malignant tissues is more significant than that of neighboring tissues (Dumont-Leblond et al., 2021). There were no significant differences between GGO and paracancerous tissue in α and β diversity (Wu et al., 2022). Various studies have found that smoking has inconsistent effects on the biodiversity of tumor tissues. The majority of studies indicate that smoking reduces the α diversity of lung tissue (Liu et al., 2018; Patnaik et al., 2021; Chen et al., 2022); BALF microorganisms are intermediate between saliva and lung cancer tissue, and the α and β diversity of saliva and BALF microorganisms are more similar in patients with high amylase of BALF, possibly due to microinhalation (Patnaik et al., 2021).

### 3.2 The correlation between alterations in intratumoral microbial communities and tumor progression

#### 3.2.1 Tissue microorganisms of GGO

Low-dose computed tomography (LDCT) is widely employed as the principal tool for lung cancer screening programs globally, and an increasing number of ground-glass opacities (GGOs) are being discovered (National Lung Screening Trial Research et al., 2011). Persistent GGO with a certain morphology is regarded as malignant and most likely an indolent, slowly progressing lung cancer (Chang et al., 2013). Consequently, it is essential to investigate the microecological characteristics of lung tissue with lung ground-glass nodules. Zhigang Wu et al. compared the differences between GGO and contralateral lower respiratory tract microorganisms and lung tissue microorganisms. The genus Rothia was abundant in the contralateral BALF, while the species Faecalibacterium prausnitzii and Bacteroides uniforms were abundant on the GGO side. GGO tissue and adjacent lung tissue have significant differences in microbiota composition at the level of class, order, family, genus, and species; interestingly, the majority of these are enriched in normal lung tissue, with an AUC of 91.05 percent (95 percent confidence interval: 81.93 to 100 percent) produced by 10 different genera, which has proven effective in detecting lung cancer (Wu et al., 2022).

#### 3.2.2 Microbial communities of the area of distant cancer tissues

Guoqin Yu et al. were the first to research the lung microbial characteristics of distant cancer tissues (n = 165) in lung cancer patients. They discovered that the microbial community of lung tissues was distinct from microorganisms in other body areas and comprised a separate bacterial colony. At the phylum level, the dominant microorganisms in distant cancer tissues were Proteobacteria, Firmicutes, Bacteroidetes, and Actinobacteria, whereas at the genus level, the dominant Bacilli were Bacillus and Bacteroides. In distant cancer tissue, the relative abundance of Thermus is greater in adenocarcinoma than in squamous cell carcinoma, whereas Ralstonia is diminished (Yu et al., 2016).

#### 3.2.3 Differentiated microbial communities of lung cancer tissue versus adjacent tissues

Compared to distant metastatic tissues, the microbial differences between lung cancer tissue and adjacent tissue provide a more accurate reflection of lung cancer.Hui Dong et al. discovered Massilia, Phenylobacterium, and Pseudoxanthomonas were enriched in lung cancer tissue; Brevibacillus, Cupriavidus, and Anaerococcus were more prevalent in neighboring tissues; and Brevundimonas, Ruminococcus, and Polaromonas were significantly different between squamous cell and adenocarcinoma carcinomas. The tumor tissues of smokers include more Massilia and Sphingobacterium and fewer Acidovorax, whereas TP53 mutation-positive tumors are rich in Acidovorax and Massilia (Dong et al., 2022). Acidovorax was also found to be more prevalent in lung cancer tissue from TP53-mutated squamous cell carcinoma patients, according to K Leigh Greathouse et al. (Greathouse et al., 2018). The recent study aimed to characterize the taxonomic profiles of the microbiota in oral saliva, cancerous, and paracancerous tissues of Chinese patients diagnosed with lung adenocarcinoma. The Shannon index of cancerous tissues (CT) was significantly higher compared to that of paracancerous tissues (PT) and saliva. The observed increased relative abundance of Promicromonosporacea and Chloroflexi, coupled with the decreased relative abundance of Enterococcaceae and Enterococcus in lung tissues, may potentially be associated with the risk of developing lung adenocarcinoma (Zhou et al., 2023).

Multiple factors, including hereditary and environmental exposures, contribute to the development of lung cancer. COPD patients are three to ten times more likely to develop lung cancer than healthy smokers (Etzel et al., 2008). Despite the fact that there are overlapping susceptibility genes (Young et al., 2008), there are also individual differences. Given that smoking is the most prevalent risk factor for lung cancer (Chaturvedi et al., 2010; Yu et al., 2014; Zhou et al., 2017), Yanhong Liu et al. collected 40 lung tissues (10 pulmonary bullae, 11 lung tumors, and 19 both lung cancer and bullae) to assess if environmental factors such as smoking influence changes in respiratory bacteria. Proteobacteria (genera Acinetobacter and Acidovorax) are more frequent in the emphysema group (Liu et al., 2018). However, the tissue sampled in this study is distant from tumor tissue, and there is a space between the cancer tissue and the sampled tissue.

#### 3.2.4 Lung cancer tissue microbes and recurrence

Lung tissue bacteria are capable of predicting the recurrence of lung cancer. The five-year survival rate for stage IA lung cancer is 83%, whereas the five-year survival rate for stage IIB lung cancer is 53% (Kay et al., 2017), and early detection of lung cancer recurrence can lessen its mortality rate. Current research on lung cancer recurrence focuses on the microenvironment of the tumor, including mutational burden (Owada-Ozaki et al., 2018), immune cell infiltration (O’Callaghan et al., 2015) and local gene expression (Planck et al., 2013; Kratz et al., 2021; Wang et al., 2021). Researchers are gradually becoming aware of the function of lung microorganisms in the occurrence and progression of lung cancer; thus, it is vital to investigate the association between early lung cancer recurrence and lung bacteria. Santosh K. Patnaik et al. compared the lung microorganisms of saliva, alveolar lavage fluid, and tissue in patients with stage I NSCLC and performed RNA sequencing on lung tissue. They discovered that Delftia and Bifidobacterium, two salivary bacteria, rose considerably in recurrent patients. Staphylococcus was more common in the lung cancer tissues of relapsed patients, but Bacillus and Anaerobacillus were more common in non-recurrent patients. There were no significant differences between the two groups in non-cancerous tissues, but there were differences in 19 genera in the BALF sample. Phingomonas, Psychromonas, and Serratia genera increased in the lung cancer recurrence group, whereas Cloacibacterium, Geobacillus, and Brevibacterium genera decreased. Lower respiratory bacteria are capable of identifying NSCLC patients with surgical recurrence, as indicated by the area under the curve value of 0.77 (Patnaik et al., 2021).

Brandilyn A. Peters et al. investigated the relationship between microbiome and recurrence in tumor and distant cancer tissue specimens (39 lung and 41 distant cancer tissues) from 46 individuals with NSCLC at stage II. There was no difference in α diversity and β diversity between cancerous and noncancerous tissues discovered. However, both α-diversity and β-diversity of the microbiota in lung cancer tissues were found to be associated with disease-free survival (DFS). In contrast, the α-diversity and β-diversity of the microbiota in adjacent tissues showed no association with DFS, recurrence-free survival (RFS), or overall survival (OS). A high abundance of species Pseudomonadales and Actinomycetales and Marmoricola aurantiacus in lung cancer tissues was negatively associated with disease-free survival (DFS). In contrast, high abundance of Bacteroides and Clostridia and orders Bacteroidales and Clostridiales in distant cancer tissues were negatively associated with survival. Survival was favorably linked with ASV of Alphaproteobacteria and Betaproteobacteria, Burkholderiales, Neisseriales, and Mycobacterium vaccae. Marmoricola aurantiacus is abundant in lung cancer tissue from individuals who have a poor prognosis (Peters et al., 2022).

Ock-Hwa Kim et al. evaluated the relationship between microbes and prognosis (recurrence) of lung cancer tissues of various pathological types and discovered that in the tissues of patients with relapsed NSCLC, the genus Stenotrophomonas accounted for the vast majority, whereas H. influenzae was enriched in patients with squamous cell carcinoma (Kim et al., 2022). Gram-positive bacteria are significantly elevated in lung adenocarcinoma, and a high bacterial load in tumors combined with increased iNOS expression is a favorable prognostic symptom factor. In contrast, a high bacterial load in tumors combined with increased FOXP3^+^ cell numbers is an indicator of a poor prognosis (Kovaleva et al., 2020).

Brandilyn A. Peters et al. demonstrated for the first time the association between normal lung tissue and lung cancer prognosis, discovering that the diversity and overall microbial composition of normal tissue were associated with decreased recurrence-free survival and disease-free survival. The more prosperous family Koribacteraceae was associated with improved recurrence-free and disease-free survival in normal tissues. Still, the Bacteroidaceae, Lachnospiraceae, and Ruminococcaceae families were inversely associated with recurrence-free survival. The diversity and composition of tumor tissue are not associated with relapse-free survival. The richness and diversity of tumor tissue are lower than that of paired normal tissue (Peters et al., 2019).

### 3.3 The association between intratumoral microbiota and factors like IAP, smoking, and family inheritance

Y. Chen et al. compared the microbiome of lung cancer patients affected by familial lung cancer (FLC) and indoor air pollution (IAP). Additionally, they describe potential links between host gene expression patterns and their microbiome. The research revealed that smoking and IAP significantly reduced the biodiversity of specific OTUs, particularly in normal lung tissue. Enhanced microorganisms include Sphingomonas and Sphingopyxis, which can decompose pollutants but can also induce epithelial injury and promote chronic inflammation. RNA sequencing data emphasize the IL17, Ras, MAPK, and Notch pathways in the FLC and IAP groups, which are associated with carcinogenesis and reduced immune function (Chen et al., 2022).

## 4 The interaction between lung microbiota and immune therapy

Only approximately 20% of patients with NSCLC derive therapeutic benefits from the anti-PD-1 antibody nivolumab. Hence, the identification of more accurate predictors becomes imperative. In their study, Chufeng Zhang et al. examined and compared the differences and correlations in gut microbes and salivary microorganisms following PD-1/PD-L1 immunotherapy. Interestingly, they observed no significant difference in respiratory microbial alpha diversity between the effective (R) and ineffective (NR) groups. However, there were notable variations in beta diversity and the abundance of Streptococcus (a respiratory flora) was found to be associated with progression-free survival (PFS) (Zhang et al., 2021). In terms of sputum α diversity, no significant difference was observed between immunotherapy-responsive and non-responsive patients, although a significant difference was noted in terms of β diversity (Zhang et al., 2021).

Gaining insight into the relationship between lower respiratory tract bacteria and therapeutic efficacy can enhance our understanding of potential markers that accurately predict the effectiveness of clinical immunotherapy. Previous studies have established a connection between alterations in lung microorganisms and immune evasion mediated by PD-L1-dependent Treg cells (Gollwitzer et al., 2014). Hye Jin Jin Jang et al. investigated the correlation between microorganisms in lung BALF and PD-L1 expression, as well as the association between microbes and the efficacy of immunotherapy. Their findings revealed a higher prevalence of Veillonella dispar (Firmicutes) in the BALF of patients exhibiting high PD-L1 expression, while Neisseria (Proteobacteria) was more commonly found in the BALF of patients with low PD-L1 expression. The immune response group had a higher abundance of Bacteroidetes and a lower abundance of Proteobacteria (Masuhiro et al., 2022). Conversely, the non-response group exhibited higher levels of the phyla Proteobacteria and Bacteroidetes, as well as the genera Haemophilus and species Haemophilus influenzae and Neisseria perflava. Additionally, the non-response group showed a lower presence of Firmicutes.

Hye Jin Jang et al. studied the lower respiratory tract microorganisms in 84 patients, including 59 patients with low PD-L1 expression and 25 patients with high PD-L1 expression. Their findings revealed a higher prevalence of Veillonella, specifically the species V. dispar, in the immune response group (Jang et al., 2021). Consequently, further research should prioritize investigating the mechanisms associated with the genus Veillonella in the immune response.

The responder group to immune checkpoint inhibitors (ICIs) exhibited higher α-diversity compared to the non-responder group, while there was no difference in β-diversity between the two groups (Masuhiro et al., 2022). Hye Jin Jang et al. also reported that there were no significant differences in α- and β-diversity between the two groups with high and low PD-L1 expression levels (Jang et al., 2021).

## 5 Potential mechanisms through which commensal microorganisms could impact the host’s immune system in relation to lung cancer development

**Figure 2:**
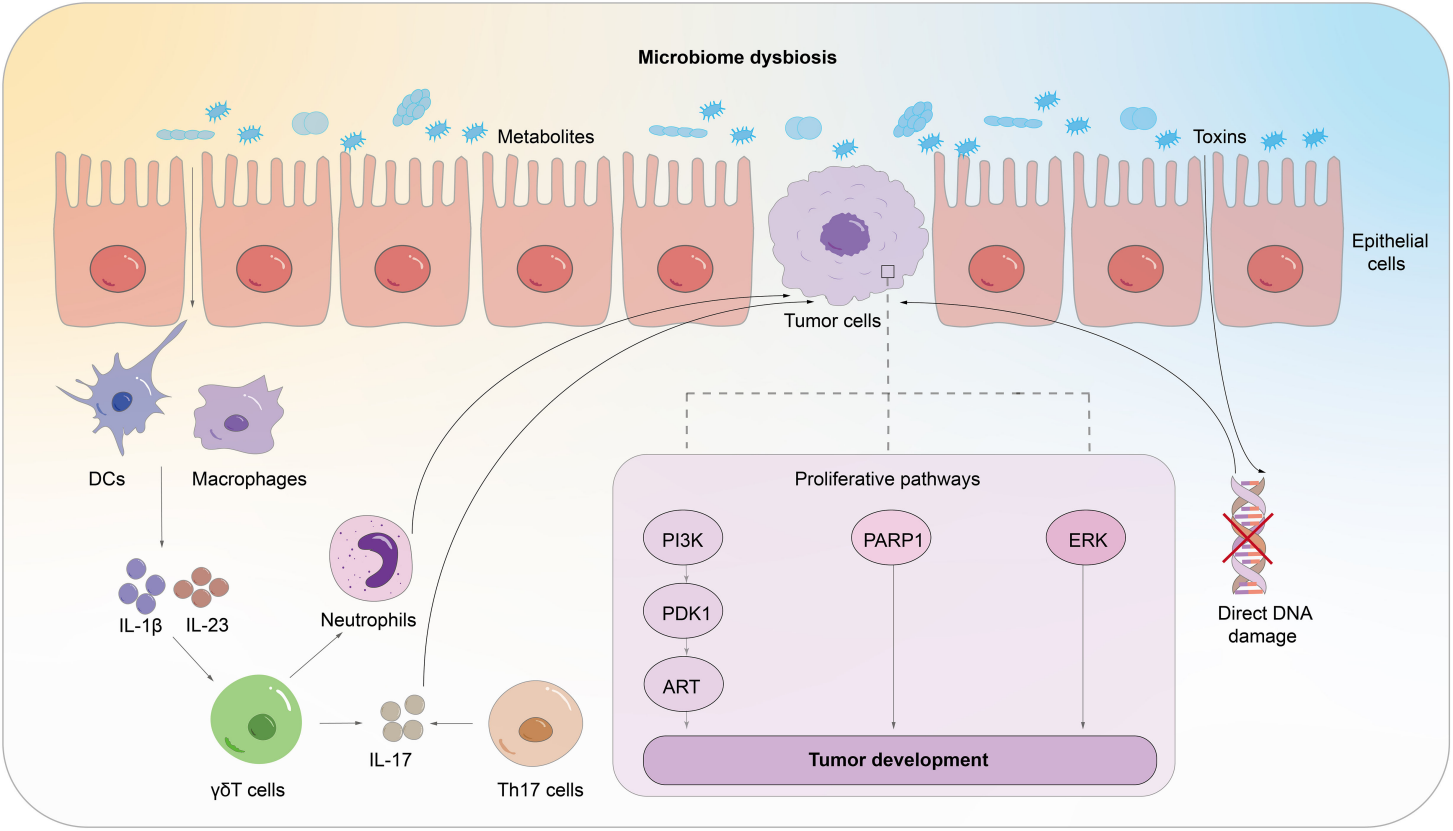
The potential mechanism through which commensal microorganisms could impact the host’s immune system in lung cancer pathogenesis. The microbiota generates cytotoxicity which induces host cell DNA damage, abnormal activation of proliferation and transformation pathways, and aberrant immunological pathways.

There is growing evidence that symbiotic microbial communities of the host are linked to the onset, progression, and therapeutic efficacy of cancer. The current literature focuses on the gut microbiome, but how the pulmonary symbiotic microbiota and the distal gut microbiota are engaged in the malignant transformation of lung cancer cells and how they simultaneously manage the balance between pro-tumor inflammation and anti-tumor immunity are not well understood. We will comprehensively overview the potential immune mechanisms involved in commensal microorganisms across four important topics (**Figure 2**).

### 5.1 The microbes inducing DNA damage and genomic instability

Genotoxins and metabolites produced by bacteria possess the capability to directly inflict damage upon the host’s DNA and instigate genomic instability via the generation of reactive oxygen or nitrogen species and the activation of innate immune receptors. When the cumulative effects of such damage exceed the host’s self-regulation capacity, it can lead to the carcinogenic impacts (Espinoza and Minami, 2018; Goto, 2020; Goto, 2022). Studies have shown that reactive oxygen species produced by Porphyromonas, hydrogen sulfide produced by Clostridium cholephilum, and superoxide dismutase produced by symbiotic bacteria can cause genomic instability and increase susceptibility to lung cancer (Attene-Ramos et al., 2006; Carbonero et al., 2012). Additionally, microbial dysbiosis can lead to increased reactive oxygen species associated with DNA damage (Mao et al., 2018).

The enrichment of bacteria such as Massilia and Acidovorax in the pulmonary system is consistent with the trends observed in DNA recombination and repair pathways (Dong et al., 2022). Folate produced by Lactobacillus and Bifidobacteria promotes the production of 6-methyltetrahydrofolate, which influences DNA methylation (Kothapalli et al., 2005; Hassan and Zempleni, 2006; Rossi et al., 2011; Zempleni et al., 2012). These studies demonstrate that microorganisms can directly or indirectly contribute to DNA damage or genomic instability.

### 5.2 Activation and regulation of the innate immune system in response to microbial influence

Microbial dysbiosis can lead to the activation of the host’s innate immune system and sustained chronic inflammation through the interaction of microbial-associated molecular patterns (MAMPs) with pattern recognition receptors (PRRs) (Evavold and Kagan, 2019; Fitzgerald and Kagan, 2020; Li and Wu, 2021). Antigen-presenting cells, such as alveolar macrophages, dendritic cells, and epithelial cells, can recognize microbial stimulation through PRRs, triggering the expression of immune-related genes and initiating innate and adaptive immune responses (Sellge and Kufer, 2015). Specifically, the activation of Toll-like receptors (TLRs) by microbial products induces the activation of alveolar macrophages and neutrophils (Chang et al., 2014; Segal et al., 2016; Jin et al., 2019). Studies have also demonstrated the upregulation of TLR-4 and TLR-9 expression in lung cancer tissue (Zhang et al., 2009; Goto, 2022). Stimulation of TLR4 using heat-inactivated Escherichia coli has been shown to activate the p38 MAPK and ERK1/2 signaling pathways, leading to adhesive, migratory, and metastatic behavior of non-small cell lung cancer (NSCLC) cells *in vivo* (Chow et al., 2015).

A reduction in microbial diversity hampers the stimulation of antigen-presenting cells, thereby impeding immune responses against tumor antigens (Bingula et al., 2017). Moreover, bacterial overgrowth triggers excessive mucosal immune responses, leading to pro-inflammatory reactions. These reactions, often mediated by the Th17 mechanism, can result in uncontrolled cell proliferation. Excessive activation of the innate immune system results in an expansion of regulatory T cells (Tregs) and M2 macrophages. These immune cells release various molecules, including prostaglandin E2 (PGE2), transforming growth factor-beta (TGF-B), and interleukin-10 (IL-10), which are known for their immunosuppressive properties. Additionally, there is an upregulation of programmed death-ligand 1 (PD-L1), contributing to immune tolerance and tumor evasion (Soroosh et al., 2013; Hussell and Bell, 2014).

### 5.3 Activation and functional regulation of the adaptive immune system in response to microbes

C.Jin et al. noted that pulmonary symbiotic microorganisms can stimulate the expression of IL-1β and IL-23, thereby inducing the proliferation of Vγ6^+^ Vδ1^+^γδ T cells that produce IL-17 and other activated molecules, promoting inflammation and tumor cell proliferation (Jin et al., 2019). Additionally, excessive bacterial overgrowth leads to overactivation of the adaptive immune system and proliferation of Th17 cells, contributing to the occurrence and progression of lung cancer (Chang et al., 2014).

Research has established that certain bacteria present in non-small cell lung cancer (NSCLC) are positively correlated with the presence of Th17 and Th1 cells. This correlation suggests a potential link between the microbiome and immune response in NSCLC (Ma et al., 2017). Pasteurella is significantly positively correlated with cytotoxic CD8+ tumor-infiltrating lymphocytes (TILs) and negatively associated with M2 macrophages. However, Coriobacteriaceae is significantly positively correlated with M2 macrophages and negatively correlated with CD8+ TILs (Zheng et al., 2020). Dysbiosis can trigger the expression of PD-L1 on CD11b^+^CD103^-^ dendritic cells in the lungs and modulate the activation of regulatory T cells (Gollwitzer et al., 2014).

Moreover, short-chain fatty acids (SCFAs) as microbial metabolites (Segal et al., 2017) induce the upregulation of the forkhead box P3 (FoxP3) in CD4^+^ lymphocytes, leading to the development of regulatory T cells (Treg) and subsequent immune tolerance (Trompette et al., 2014). However, elevated SCFAs levels in the lungs may inhibit the production of interferon-gamma (IFN-γ) by CD4^+^ and CD8^+^ T cells, resulting in T cell exhaustion and suppression of cytotoxicity against malignant cells (Segal et al., 2017).

### 5.4 The interaction between microbes and epithelial cells

The stimulation of epithelial cells by microorganisms can lead to abnormal activation of proliferation and transformation pathways, resulting in carcinogenic effects. The presence of specific microbial species in non-small cell lung cancer (NSCLC) has been found to be associated with the dysregulation of oncogenic transcriptional systems. Research findings indicate that Prevotella, Streptococcus, Veillonella, and tiny Vibrio can stimulate airway epithelial cells, activating the ERK and PI3K signaling pathways. Excitingly, Cyanobacteria-produced microcystins promote cell proliferation by inducing PARP1 overexpression (Apopa et al., 2018).

Stimulation of epithelial cells by nontypeable Haemophilus influenzae (NTHi) leads to the expression of IL17C, which in turn promotes neutrophil infiltration and chronic inflammation and facilitates tumor progression (Jungnickel et al., 2017). Furthermore, interleukin-6 (IL-6) has been shown to promote lung cancer growth by inducing inflammatory responses (Ochoa et al., 2011). Furthermore, CD36 is a crucial link between lung microbiota and cancer. It regulates microcystin processing in the alveoli, increasing PARP1 expression and promoting non-small cell lung cancer development (Apopa et al., 2018).

## 6 Conclusion

In this review, we evaluated the link between respiratory tract flora and lung cancer, focusing on the characteristics of microbial flora in different locations of lung cancer, including saliva, sputum, BALF, bronchial brushing samples, and tissues, and analyzing the relationship between microbial flora in other parts and genes, environmental factors, pathological types, stages, treatment, and prognosis. The studies on the potential role of microorganisms in the pathogenesis and progression of lung cancer were reported in this article.

So far, the following conclusions can be drawn: Firstly, independent of locations, the microbial composition of lung cancer patients differed considerably from that of healthy individuals, indicating that the microbial flora has the potential to act as a biomarker for the diagnosis and prognosis of lung cancer. Secondly, microorganisms found in the lower respiratory tract, such as BALF, BWF, and tissues, are more suggestive of lung cancer. Finally, in lung cancer patients, pulmonary symbiotic bacteria directly or indirectly promote or inhibit tumor progression via the pro-inflammatory and anti-inflammatory balance, immune response dysfunction, metabolic pathway, DNA damage, and genomic instability. In conclusion, pulmonary microbiomes may be biomarkers for predicting lung cancer’s onset, progression, and prognosis. The causal association between respiratory microbes and lung cancer must be established through more mechanistic research.

The result contradicts between samples reflects the dynamic change of microbiota in carcinogenesis, suggesting that specific bacteria may play different roles at different time points and different host locations (Ramirez-Labrada et al., 2020), which may also be due to the small sample size, regional, dietary, and other factors to resulting in the identical examination of several bacteria. To determine the microbiological characteristics of lung cancer, large-scale microbial investigations on lung cancer with control of intervention factors will be required in the future.

Nowadays, lung cancer is the most lethal tumor, placing a substantial burden on the lives and economies of people all over the world. Therefore, research into the complete pathophysiology of lung cancer, the quest for new therapeutic techniques, and the development of specialized therapeutic medications remain essential for the prevention and treatment of this condition. Due to the intense research interest in the mechanism of action of intestinal microbes on the incidence of colorectal cancer and the effect of pharmacological therapy, the association between pulmonary microbial flora and lung cancer has been increasingly recognized, but it remains a descriptive study. Future studies on the mechanism, prevention, and therapy of microbial involvement are urgently required. Numerous confirmatory cellular and animal experiments must be conducted, which will likely open up new avenues for researching lung cancer disease mechanisms.

## Author contributions

JC: Writing – original draft, Writing – review & editing. LZ: Funding acquisition, Writing – review & editing. HW: Supervision, Writing – review & editing.
